# Seasonal Variation in Vitamin D in Association with Age, Inflammatory Cytokines, Anthropometric Parameters, and Lifestyle Factors in Older Adults

**DOI:** 10.1155/2017/5719461

**Published:** 2017-09-14

**Authors:** Leticia Elizondo-Montemayor, Elena C. Castillo, Carlos Rodríguez-López, José R. Villarreal-Calderón, Merit Gómez-Carmona, Sofia Tenorio-Martínez, Bianca Nieblas, Gerardo García-Rivas

**Affiliations:** ^1^Centro de Investigación en Nutrición Clínica y Obesidad, Escuela de Medicina, Tecnologico de Monterrey, 64849 Monterrey, NL, Mexico; ^2^Centro de Investigación Biomédica, Hospital Zambrano Hellion, Tecnologico de Monterrey, 66278 San Pedro Garza-García, NL, Mexico; ^3^Cátedra de Cardiología y Medicina Vascular, Escuela de Medicina, Tecnologico de Monterrey, 66278 San Pedro Garza-García, NL, Mexico

## Abstract

Vitamin D deficiency is present even in sunny regions. Ageing decreases pre-vitamin D production in the skin and is associated with altered cytokine profile. We performed a multivariate analysis considering lifestyle factors, anthropometric, and inflammatory markers according to seasonal variation in Mexican healthy older adults. The same cohort was followed during 12 months. Vitamin D deficiency/insufficiency was found in 91.3% of the subjects despite living in appropriate latitude (25°40′0″N). 25(OH)D levels remained below <30 ng/mL through all seasons. Vitamin D deficiency did not correlate to sun exposure or dietary intake. Gender was the strongest associated factor, explaining a variance of 20%. Waist circumference (WC) greater than 88 cm was a risk factor for vitamin D deficiency. Age (>74 years) combined with WC (>88 cm) and BMI (>32.7) showed a high probability (90%) of vitamin D deficiency. Remarkably, an increase in one centimeter in WC decreased 25(OH)D by 0.176 ng/mL, while an increase in one point BMI decreased 25(OH)D by 0.534 ng/mL. A cutoff point of 74 years of age determined probability of vitamin D hipovitaminosis. Vitamin D deficiency was correlated with TNF-*α* serum levels, possibly increasing the susceptibility of older adults to a proinflammatory state and its related diseases.

## 1. Introduction

The main source of Vitamin D comes from sun exposure of the skin, from 7-dehidrocholesterol in response to ultraviolet B radiation (UVB), to further be metabolized in the liver to 25-hydroxyvitamin D 25(OH)D which is the metabolite used to assess vitamin D status. It requires further metabolism by the 1-alpha hydroxylase (CYP27B1) in the kidneys to produce the biologically active form 1,25-dihydroxyvitamin D (1,25(OH)2D3). In addition to the kidneys, many other tissues and cells throughout the body, including immune cells, express CYP27B1 and are therefore capable of regulating their own 1,25(OH)2D3 local concentration [1].

While the CYP27B1 in the kidneys is regulated by parathyroid hormone (PTH), fibroblast growth factor-23 (FGF-23), and 1,25(OH)2D3, its production in the immune system cells is driven by inflammatory conditions, either directly by the presence of cytokines [2] or in response to activation through the vitamin D receptor (VDR). Activated VDR binds response elements on DNA that are associated with antimicrobial [3] and immune regulatory functions [4]. Thus, vitamin D deficiency might result in the impaired function of immune cells [5] and cytokine imbalance [6, 7]. It has also been observed that sufficient 25(OH)D levels are related to increasing concentrations of IL-4 and IL-10 and to low levels of proinflammatory cytokines such as IL-1, IL-6, IL-12, IL-17, IL-23, and IFN-*γ* [3, 8]. Aging is associated with changes in the immune system, with a decline in the number of naïve T cells in favor of an increase in memory T cells, and with a relative increase of Th2 cell versus Th1 cells [9]. These shifts in the T cell population change the cytokine expression profile. Furthermore, there is a decline in the level of androgens and estrogens, which have been linked with suppression of IL-6, a proinflammatory cytokine [9]. In this regard, vitamin D deficiency has been linked with several diseases such as autoimmune disorders [10], osteoarthritis [11], and cancer [12], in which chronic inflammation might be a causative factor.

In the US, 41.6% of the population is estimated to be vitamin D deficient, with Hispanics being the second ethnic group most at risk (69.2%) behind African Americans (82.1%) [13]. In Mexico, the biggest study to date found a prevalence of 9.8% for vitamin D deficiency and of 20% for insufficiency [14]. However, a more recent survey revealed a contradictory result, indicating a vitamin D deficiency prevalence of 43.6%, while an additional 46.8% were vitamin D insufficient [15].

Some studies have found that vitamin D levels increase in summer and decrease in winter [16–18] due to dependency of vitamin D on sunlight. Studies have also shown that this seasonal variation might depend on latitude, since it has been found that vitamin D production is greater on latitudes close to the equator [19]. However, vitamin D deficiency has even been reported in sunny regions [20].

This deficiency has been linked to many factors. For instance, skin pigmentation has a strong effect on vitamin D status, since it reduces the UVB radiation that effectively reaches the skin [21]. In the same way, sunscreen use decreases vitamin D production [22]. On the other hand, obesity is a risk factor. It has been proposed that this is due to fatty tissue uptake of vitamin D, reducing its bioavailability [23]. BMI and WC in particular have been negatively associated with vitamin D levels and greater prevalence of deficiency [18, 24, 25]. Regarding age, it has been proposed that vitamin D deficiency in the elderly can be attributed to a decrease in the skin capacity to produce vitamin D due to ageing, from a lack of exposure to sunlight, or from a deficient dietary intake [26].

However, there are scarce results examining vitamin D deficiency and its association with anthropometric, inflammatory, and lifestyle factors in the same cohort of patients through 12 months. There are also, to our knowledge, no studies of this type searching for seasonal changes in the same cohort in regions at latitude 25°40′0″N, where sunlight is adequate throughout the year. Therefore, the aim of the current study is to evaluate a 12-month follow-up seasonal variation of vitamin D status and its association with proinflammatory cytokines, anthropometric parameters, and lifestyle factors in Mexican healthy older adults.

## 2. Materials and Methods

### 2.1. Study Population

A longitudinal study was done in a sample of healthy adults older than 55 years old (55–86 yo) in the city of Monterrey, in the northeastern part of Mexico (latitude 25°40′0″N). The same cohort of patients (*n* = 23) was followed for the duration of 12 months; all parameters assessed were evaluated every three months according to the seasonal variation. Inclusion criteria required subjects to be older than 55 years old, be generally healthy, and be community living independent. Exclusion criteria included renal disease, hepatic disease, diabetes mellitus, hypertension, cardiovascular diseases, use of corticosteroids, oral contraceptives, anticonvulsant medications, gastrointestinal malabsorption diseases, gastrointestinal resections (bariatric surgery), consumption of supplements containing vitamin D, the use of sunscreen, living in nursing homes, or being institutionalized. Written informed consent was obtained from all of the subjects. Approvals by the Ethics and Research Committees of the School of Medicine, TEC de Monterrey, and by the Secretariat of Health were obtained. This study was registered in clinical trials with the following code: NCT02087683. Each subject was evaluated every three months according to each season of the year.

### 2.2. Vitamin D Intake and Sun Exposure

Subjects answered a previously validated face-to-face questionnaire [27], which was applied by a registered dietitian. The questionnaire included weekly consumption of foods rich in vitamin D such as milk, yogurt, cheese, fish, egg yolk, fortified fruit juice, margarine, and cereals, as well as vitamin supplements. Subjects were shown portion size kits to determine the number of portions per day and the number of days per week each food was consumed. In addition, subjects were screened for sun exposure, specifically between 11 : 00 AM and 3 : 00 PM in minutes per day and days per week. The use of sunscreen was also included in this screening, as an exclusion criteria. Skin phototypes were assessed according to Fitzpatrick's classification. None of the patients were outdoor workers.

### 2.3. Anthropometric Measurements

The anthropometric variables were evaluated every three months, according to seasonal variation at the moment of the blood withdrawal for vitamin D and the other laboratory variables. The anthropometric parameters measured were weight, height, waist circumference (WC), and percentage of body fat (BF%) measured by bioimpedance (standardized TANITA 350; BMI was calculated as kg/m^2^) according to standardized protocols [28].

### 2.4. Vitamin D, Metabolic Assessment, and Cytokines

Blood samples were taken from each subject to obtain a total of four samples per patient, one sample for each season of the year. Serum 25(OH)D and parathyroid hormone were measured by chemiluminescence and calcium by spectrophotometry within four hours of venipuncture. Additional blood samples were centrifuged to obtain serum and plasma. Serum and plasma samples were frozen at **−**80°C and used to measure total calcium, phosphorous, parathyroid hormone, and cytokine profile. Cytokine profile was performed in serum samples using the Legendplex Human Inflammation panel for a multianalyte cytometric assay (BioLegend, San Diego, CA, USA). The cytokine capture beads measured were IL-1*β*, IFN-*α*, IFN-*γ*, TNF-*α*, MCP-1, IL-6, IL-8, IL-10, IL-12p70, IL-17A, IL-18, IL-23, and IL-33. Each experiment was performed in triplicate, as per manufacturer's instructions. Data were collected on a flow cytometer FACSCanto II (Becton Dickinson, USA). The analyte concentration was calculated using the standard curve provided, and serum concentrations of all cytokines were determined using the Legendplex software (BioLegend). According to the guidelines of the Endocrine Clinical Society, vitamin D deficiency was defined as a level of serum 25(OH)D < 20 ng/mL, vitamin D insufficiency between >20 and <30 ng/mL, and vitamin D sufficiency as >30 ng/mL [29].

### 2.5. Statistical Analysis

An exploratory analysis using principal component analysis (PCA) was performed for continuous variables, considering only subjects who presented no missing values. Pearson correlations and hierarchical clustering analysis were used to assess the relation among cytokines, as well as between 25(OH)D concentration and anthropometric parameters. Confirmation of correlations of 25(OH)D concentration was done using uni- and multivariate linear regressions, blocking by sex. A conditional inference tree [30] was generated using anthropometric parameters to obtain ranges of prediction for 25(OH)D levels.

Finally, a linear mixed effects model was performed, evaluating the effect of seasonal variation in 25(OH)D and cytokines concentration, considering age, sex, and individual as random effects, using maximum likelihood to calculate *p* values [31] and Tukey all pair comparisons for post hoc grouping. Statistical analyses were done in the R platform (R Core Team) using the packages lme4 [32], multcomp [33], and partykit [34], along with the included stats library.

## 3. Results

### 3.1. Demographic, Anthropometric, and Lifestyle Parameters

As a description of the studied population, Table 1 presents the demographic and anthropometric characteristics of the participants. A total of 23 patients were recruited, with a mean age of 68.8 years (range 55–86 years) and a similar number of male (10) and female (13) participants. According to the Fitzpatrick phototyping scale, the majority had type IV skin. Subjects had a mean BMI of 29 (20.1–46.6), a WC of 99.8 cm (78–121), and a BF% of 35.2 (17.5–51.3). The mean vitamin D intake was 112 IU (0–391.4) per day. The most common food source of vitamin D was milk, followed by fatty fish and cheese. The mean sun exposure occurred between 11 : 00 AM and 3 : 00 PM, equaling 82.3 min (0–840) per week.

### 3.2. Unsupervised Multivariate Analysis

**Figure 1:**
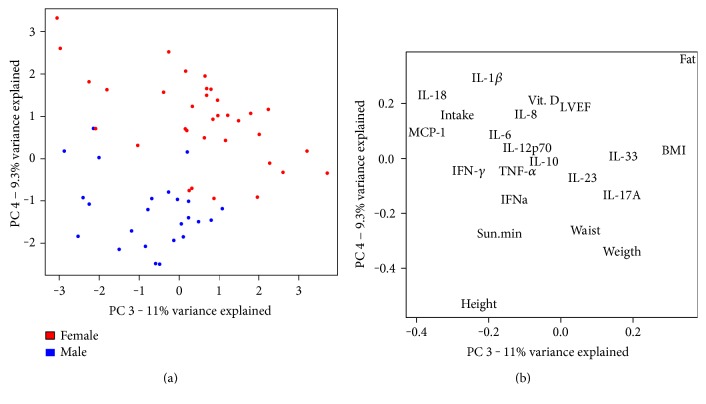
Principal component analysis of the continuous variables. Score plot (a) showing the separation of the female (red) and male (blue) population; and loadings plot (b) showing the variables responsible for the separation.

Gender was the parameter that could explain the most variance (20%, principal components 3 and 4; Figure 1(a)). The main anthropometric and clinical parameters affecting this separation were body fat, BMI, and 25(OH)D levels, which were higher in women. Height, WC, weight, and minutes of sun exposure were higher in men (Figure 1(b)). Thus, blocking by gender was necessary and was expected to attenuate sampling effects due to these parameters. None of the other analyzed factors appeared to have an effect on the data set.

### 3.3. Anthropometric Parameters and Age but Not Lifestyle Factors Are Related to Vitamin D Levels

**Figure 2:**
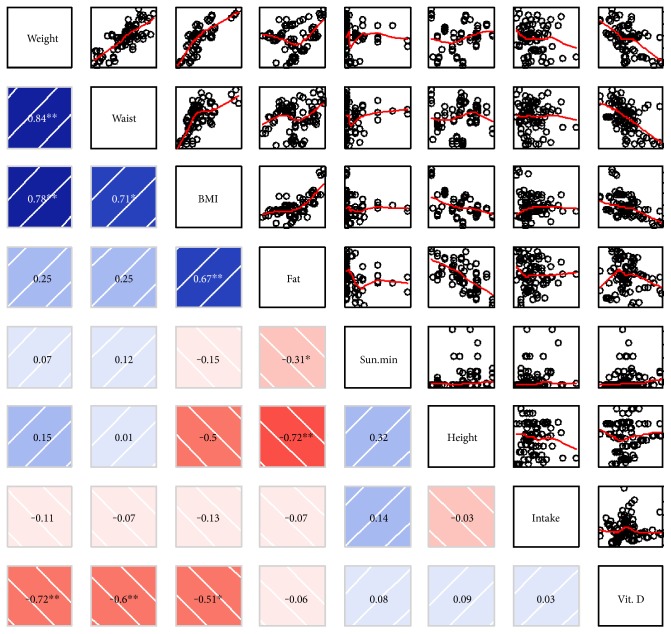
Correlation of anthropometric parameters and 25(OH)D. Upper panels show the raw data (circles) and locally weighted smoothing (red line) and lower panels show the Pearson correlation coefficient (*r*). Significant correlations are shown in bold (^∗^*p* < 0.05, ^∗∗^*p* < 0.01).

**Figure 3:**
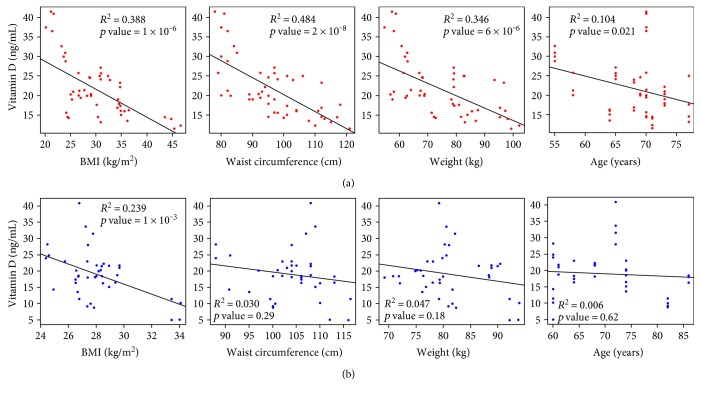
Individual regressions, separated by sex. Linear regressions between 25(OH)D levels and BMI, WC, weight, and age from left to right are shown as a black line in female (a) and male (b) populations. Coefficients of determination (*R*^2^) and *p* values are shown inside the plots in bold for significant correlations.

As shown in Figure 2(), only one cluster with three parameters showed a significant (*p* < 0.01) negative correlation with 25(OH)D concentration, namely, weight, WC, and BMI, which were highly correlated to each other. Interestingly, neither dietary vitamin D intake nor sun exposure affected 25(OH)D levels. Focusing on these correlated parameters, a general linear regression was performed, blocking by sex, which revealed that indeed there was a significant negative correlation between 25(OH)D levels and WC (*p* < 0.001), BMI (*p* < 0.001), and weight (*p* < 0.001). We found a significant negative correlation between vitamin D concentration and age for women but not for men (Figure 3()). When analyzing data separately, only BMI remained a significant (*p* < 0.01) predictor in the male population, with a determination coefficient of 0.24 (data not shown). Interestingly, WC explained 48.4% of the variation in 25(OH)D levels in women (*p* < 0.001), while it was not significant in men (*p* > 0.2) when analyzed separately. A multivariate lineal model, using sex as a covariate, showed that in women an increase in one centimeter in WC translates to a decrease in 25(OH)D concentration of 0.176 ng/dL. An increase in one BMI unit decreased vitamin D by 0.534 ng/dL (Table 2).

**Figure 4:**
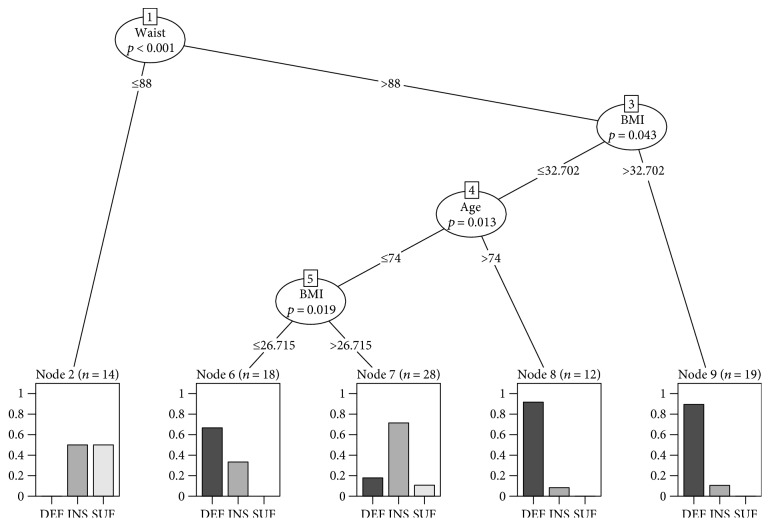
Decision tree relating vitamin D levels and anthropometric parameters. Variable name and *p* values are shown inside the bubbles, and cut-off values at the corresponding line. Plots at the bottom show the sample distribution, when rules are followed: for example, of all patients with WC >88 cm and BMI >32.702 kg/m^2^ (rightmost plot), 90% have vitamin D deficiency and 10% have insufficiency. DEF = deficiency; INS = insufficiency; SUF = sufficiency; WC = waist circumference; BMI = body mass index.

In our effort to generate predictors of 25(OH)D levels using anthropometric parameters, a conditional inference tree was used to generate simple classification rules to aid diagnosis (Figure 4()). The model, which first considered all anthropometric measures, only found WC, BMI, and age to be of relevance in classifying patients by vitamin D level; this agreed with the correlations and linear model. The decision tree correctly classified 73.7% of the patients according to vitamin D status, with only 8% of false positives for sufficiency (Table 3). The main predictor was WC. A WC exceeding 88 cm was considered as a risk factor for vitamin D insufficiency and deficiency. This WC combined with a BMI greater than 32.702 indicated an extremely high probability (90%) of having vitamin D deficiency. On the other hand, a cutoff point of 74 years of age is a determinant of vitamin D status. A BMI ≤ 32.7 and an age > 74 years also predict a 90% probability of vitamin D deficiency and 10% of insufficiency, while an age < 74 years is a predictor of 70% insufficiency versus 20% deficiency status (Figure 4()).

### 3.4. Vitamin D Seasonal Variations

**Figure 5:**
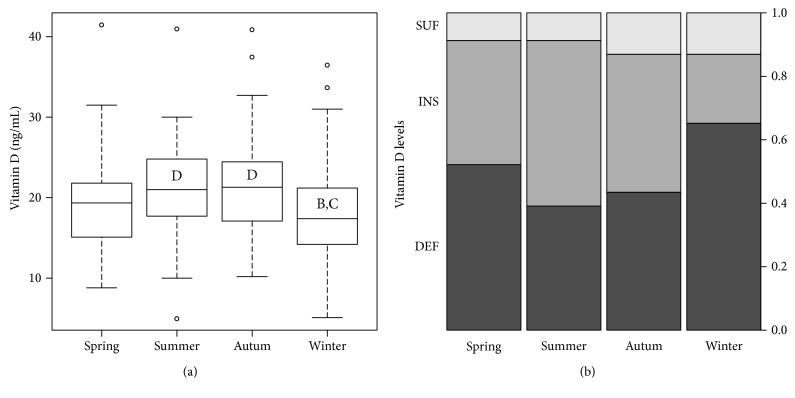
Seasonal variation of vitamin D. (a) 25(OH)D concentrations in the four seasons; the bold line represents the median, the box encloses the first and third quartiles, and the whiskers, the minimum and maximum value; points are outliers (still considered in the statistical analysis). Letters denote statistical difference between group resulting from Tukey's all pair comparisons (*p* < 0.05). (b) Prevalence of the different 25(OH)D levels and vitamin D status during the four seasons: deficiency, dark gray; insufficiency, gray; sufficiency, light gray.

The results shown in Figure 5(a) indicate that winter is the season in which the subjects had the lowest levels of 25(OH)D (18.8 ± 7.5 ng/mL), with concentrations recovering in spring (20 ± 7.3 ng/mL), to reach maximum levels in both summer (21.1 ± 7.3 ng/mL) and autumn (21.9 ± 7.5 ng/mL). 25(OH)D concentrations were significantly lower in winter compared to summer (*p* < 0.05) and autumn (*p* < 0.001).  Figure 5(b) shows the prevalence of vitamin D deficiency and insufficiency, which follows a trend agreeing with the previous results, with more than 60% of patients having a deficiency of vitamin D in winter, compared to less than 40% having a deficiency in summer. During winter and autumn, 87.5% of the subjects had 25(OH)D levels <30 ng/mL, while in summer and spring, 91.3% had.

### 3.5. Cytokine Seasonal Variation

**Figure 6:**
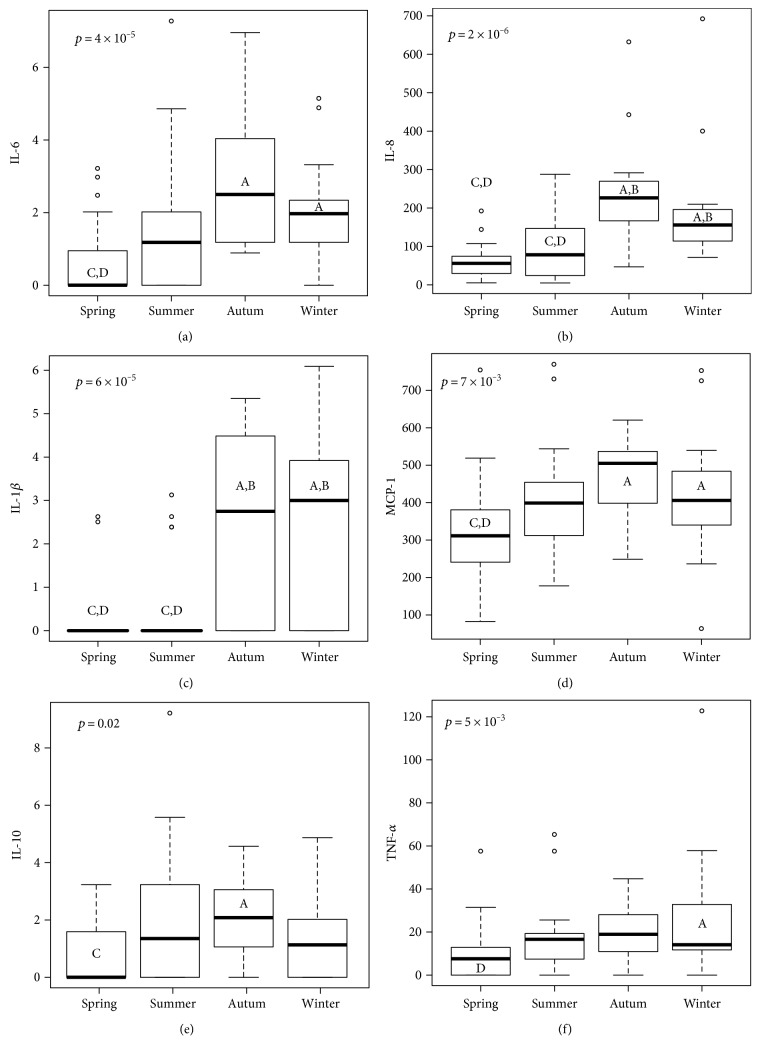
Seasonal variation of the cytokine panel. Concentrations of IL-6 (a), IL-18 (b), IL-1*β* (c), MCP-1 (d), IL-10 (e), and TNF-*α* (f) during the four seasons. The bold line represents the median, the box encloses the first and third quartiles, and the whiskers, the minimum and maximum value; points are outliers (still considered in the statistical analysis). Letters denote statistical difference between groups resulting from Tukey's all pair comparisons (*p* < 0.05).

**Figure 7:**
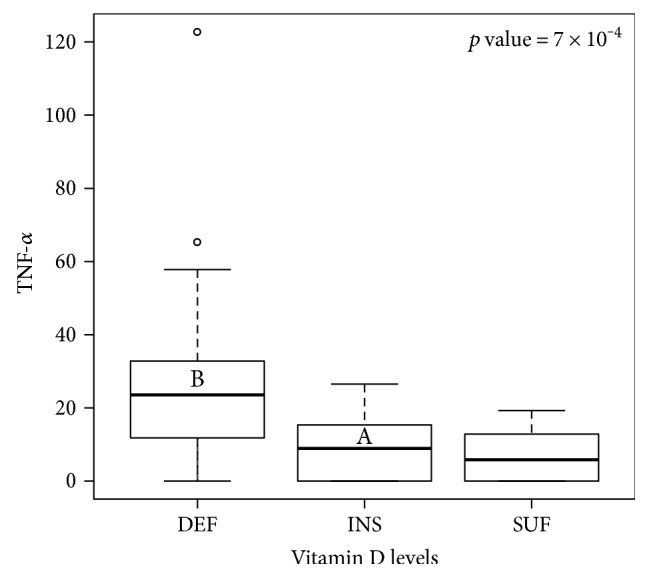
Relation of TNF-*α* with vitamin D. Concentrations of TNF-*α* are plotted against the 25(OH)D level. The bold line represents the median, the box encloses the first and third quartiles, and the whiskers, the minimum and maximum value; points are outliers (still considered in the statistical analysis). Letters denote statistical difference between groups resulting from Tukey's all pair comparisons (*p* < 0.05). DEF = deficiency; INS = insufficiency; SUF = sufficiency.

**Figure 8:**
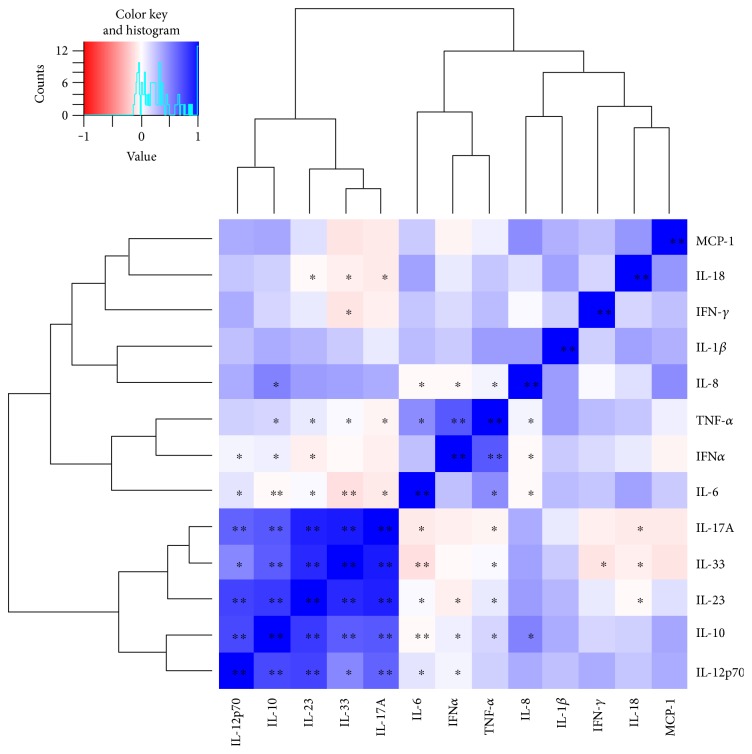
Correlations among cytokines. Pearson correlation coefficients are shown in the heatmap as a color gradient, from red (*r* = −1) to white (*r* = 0) to blue (*r* = 1). Significant correlations are shown as ^∗^*p* < 0.05, ^∗∗^*p* < 0.01.

After blocking by gender, the panel of cytokines (IL-1*β*, IL-6, IL-10, IL-18, MCP1, and TNF-*α*) significantly varied with the season, considering age, sex and individual as random effects. As shown in Figure 6, all of these cytokines have the highest discernible levels in autumn, winter, or both, while the lowest discernible concentration is invariably in spring. This variation is independent from 25(OH)D concentrations, except for TNF-*α*, the levels of which are exacerbated in patients with vitamin D deficiency (Figure 7). A hierarchical clustering was performed for the cytokine measurements, and the results of which are displayed in Figure 8. Cytokines are therefore grouped in two main clusters, the bigger being a set of five significantly (*p* < 0.01) positive correlated cytokines: IL-17A, IL-33, IL-23, IL-10, and IL-12p70. The second cluster conformed by TNF-*α*, IFN-*α*, and IL-6 correlated among them (*p* < 0.05), but not with 25(OH)D levels. The remaining cytokines are not significantly correlated with one another, although IL-18 is correlated to the main cluster via positive, significant (*p* < 0.05) correlations with IL-17A, IL-33, and IL-23; similarly IL-8 is significantly (*p* < 0.05) correlated to all members of the second cluster (TNF-*α*, IFN-*α*, and IL-6); and finally, IFN-*γ* is significantly correlated with IL-33. When analyzing the periodicity of proinflammatory compound expression separating by gender, some of the cytokines remained significantly different for men and for women (IL-18, IL-6, and IL-1*β*; 0.012 and 0.0002, 0.008 and 0.006, 0.003 and 0.007, resp.), while others remained significant only in men (MCP-1 and TNF-*α*; 0.044 and 0.002, resp.).

## 4. Discussion

This was a longitudinal 12-month follow-up study of the same cohort to determine vitamin D seasonal changes and their association with anthropometric parameters, lifestyle factors, and proinflammatory cytokines in an older adult Mexican population. Our results show a great prevalence of vitamin D deficiency and insufficiency across all seasons, with significantly greater prevalence of deficiency in winter compared with summer and autumn. Vitamin D levels were negatively correlated with BMI, WC, and weight, as well as with gender differences and TNF-*α* levels. While WC explained almost half of the variations in vitamin D levels in women, BMI was the second significant predictor of vitamin D. However, neither dietary vitamin D intake nor sun exposure affected 25(OH)D levels.

Vitamin D deficiency or insufficiency is very common worldwide. Our results in this sample of Mexican older adults show that 25(OH)D levels were <30 ng/mL through the four seasons with significant seasonal variations as well as in the prevalence of deficiency/insufficiency which was around 90%. 25(OH)D levels were significantly lower in winter than in summer and autumn. Other countries have also shown a high level of vitamin D deficiency, but not as high as ours. A study performed in seven different cities of Canada showed that about 60% of subjects > 35 years had levels of 25(OH)D below 30 ng/mL [16]. In Europe, vitamin D deficiency was estimated to be 40.4% where ethnic groups with more skin pigmentation were found to have higher prevalence of vitamin D deficiency [35]. In Mexico, a recent survey in an open population (≥14 years old) showed a deficiency prevalence of 43.6%, with 46.8% of the population in the insufficiency range [15]. These findings are similar to those reported in Canada and Europe, despite Mexico's more favorable latitudinal position. These results could be explained by the characteristics of our subject population (skin type IV and increased age), since as we observed, age could also be a factor for insufficiency predictor. In agreement with our findings, data from NHANES 2007–2010 found that 75% of the USA population had 25(OH)D levels below 30 ng/mL and 35% had levels below 20 ng/mL. When stratified by race, 83% of Hispanics had levels below 30 ng/mL, and 36% below 20 ng/mL [36].

### 4.1. Seasonal Variations

There are some studies that also take into consideration seasonal variations of vitamin D status; however, none of them performed a follow-up seasonal variation of the same cohort as we did. In this regard, in studies where only one sample was taken for each subject (some of them being taken in summer and others in winter), a significant seasonal variation in vitamin D status was seen in Caucasians [17] and Swedes [18], but not in subjects from Iran [37]. A study in Australia, on the contrary, found vitamin D deficiency prevalence in summer that rose in spring but not in winter [38]. The seasonal variation found in most studies might be due to the fact that during winter in the different countries, not only is there less sunlight but also the UVB rays enter the earth in a tangential position that do not reach the skin at the right angle, and pre-vitamin D production in the skin is greatly reduced [39]. Nevertheless, it does not explain why even when this study took place in a city in the northeastern part of Mexico at an appropriate latitude (25°40′0″N), the prevalence of vitamin D deficiency and insufficiency was even greater than at other sites where the latitude is not appropriate, even during sunny seasons when the maximum pre-vitamin D production is in the skin.

### 4.2. Sun Exposure, Gender, Age, and Anthropometric Parameters

In agreement with our findings, a worldwide meta-analysis found no association between latitude and serum 25(OH)D levels [40]. Nevertheless, we must also take into consideration that during winter, there are fewer people who exercise or are outdoors due to the cold weather in our city. In this sense, a cross-sectional study indicated that outdoor exercise reduced the risk of vitamin D deficiency/insufficiency, since patients who did outdoor exercise had less prevalence of hypovitaminosis D than those who did not exercise outdoors [41]. Taking this in consideration, we analyzed sun exposure and dietary intake and found that neither of them affected 25(OH)D levels in our population. The dietary vitamin D intake was about 112 IU per day, way below the recommendations of the IOM (600–800 IU; IOM2011), while the Endocrine Society Clinical Practice Guideline agrees that adults need an intake of 1500–2000 IU [29]. This could partially explain the low vitamin D levels and high prevalence of deficiency of the population, but sun exposure continues to be the main source of vitamin D.

Even though our population's sun exposure between 11 : 00 AM and 3 : 00 PM was 83.4 minutes per week, exceeding the sun exposure recommendations for UBV radiation and vitamin D production (10–15 minutes three times a week between these hours) [42], we must emphasize that most of the population was skin type IV, which lessens the pre-vitamin D production in the skin. Similar to our results, a cross-sectional study with healthy subjects aged 65 years and older concluded that differences in sun exposure do not explain the differences in vitamin D status [43].

When we performed a deeper analysis, taking into consideration other parameters that have been associated with vitamin D deficiency observed in our studied population, we found that gender explained the most variance of 25(OH)D levels, with women having higher 25(OH)D levels than men. We found a significant negative correlation between vitamin D concentration and age for women but not for men. Although there is no clear explanation for this gender difference, the fact that in women, BMI, WC, and weight had a negative correlation with vitamin D levels, while in men, only BMI did, might be related. In addition to this, women presented higher body fat and BMI, while men demonstrated higher height, WC, weight, and minutes of sun exposure. These results agree with some other studies.

In Canada, gender differences were significant, with deficiency prevalence being 30% for men and 24% for women [44]. In this regard, a large meta-analysis across the world also showed that 25(OH)D levels were higher in women than in men [40]. On the contrary, in Iranian subjects, women had slightly lower levels of 25(OH)D than men (20.6 versus 23.2 ng/mL) [37]. A large study in obese adults suggested that subcutaneous fat in women allow them to store vitamin D, which would be released when cutaneous production decreased [25]. Given that gender is a strong predictor, when we performed the analysis after blocking by gender, we found a negative correlation between 25(OH)D levels and WC, BMI, weight, and age, but not with the % of body fat. A possible explanation for the lack of correlation of vitamin D levels with body fat percentage might be that BMI, weight, and WC were correlated among themselves, while body fat percentage was not. Although most studies agree that vitamin D levels are negatively correlated with body fat percentage because of its sequestration by fat cells, one study attributes a lack of correlation to a volumetric dilution instead, which might in part explain our results (46). Another explanation might be the wide range of body fat percentage of the participants, from very low (17.5) to very high (51.3) which might have influenced the result. One more possibility would be that we assessed fat percentage but not fat mass in kg, which represents better the total adipocyte mass.

Similarly, observational studies have linked an increase in BMI with lower levels of vitamin D [24, 25]. A negative correlation between BMI and serum vitamin D levels has been found in obese [25] and in nonobese populations [23]. In another study, a significant negative correlation was found between body fat and vitamin D serum levels [45]. In this sense, a cross-sectional study showed that weight, body fat mass, and BMI were negatively correlated to 25(OH)D levels [46], as well as WC and other obesity markers [47, 48].

It has also been observed that in women, even after adjusting for BMI, ethnic group, age, and season, vitamin D remained associated with total abdominal adipose tissue, VAT, SAT, and body fat percentage. It also remained associated with VAT and body fat percentage in men [49]. Remarkably, when our data was analyzed separately, BMI was a significant predictor of 25(OH)D levels in the male population. In women, WC explained 31% of the variation in 25(OH)D levels and showed that for each centimeter increase in WC, there was a decrease of 0.176 ng/mL in the level of 25(OH)D. As well, for each one increase in BMI, concentration of 25(OH)D decreased by 0.534 ng/mL. Furthermore, the decision tree established that the main predictor of vitamin D levels is WC and that in combination with BMI and age, the degree of possibility of vitamin D deficiency can be predicted. Similar to our results, a cross-sectional study showed that weight was the variable most related to vitamin D levels, followed by body fat mass and BMI. Total weight combined with fat mass was the strongest linear fit, which explained 10.4% of the variation in vitamin D levels [46].

A retrospective study in Turkey found that the highest 25(OH)D levels were found in the age group 0–10 and the lowest in the 10–40 years old age group. The highest prevalence of vitamin D deficiency was found in the age group 20–30 years old. The authors suggested that older adults may be taking supplements. About 80% had 25(OH)D levels below 30 ng/mL from February to May, but only 58% remained with such levels in summer. A negative correlation (*p* < 0.001) was found between 25(OH)D and PTH levels [50]. However, this study did not take into account information about vitamin D supplementation nor about any lifestyle (e.g., diet, vitamin D intake, and sun exposure) or anthropometric variables. As the authors suggested, older adults might have been taking supplements, and this would be the reason why younger people had lower values. In our study, vitamin supplementation was an exclusion criteria and we did consider vitamin D intake in foods as well as sun exposure. Thus, our results are not influenced by vitamin D supplementation.

The skin content of 7-dehidrocholesterol as well as the response to UV radiation decreases with aging, resulting in a 50% decrease in the cutaneous production of pre-vitamin D [26]. Another author states that the capacity of skin production of vitamin D in a 70-year-old is reduced by 75% compared to the capacity of a young adult [51]. Based on extrapolations from other studies, it is estimated that the average adult older than 60 years living in southern (35°N) United States produces less than 600 IU/day of vitamin D in response to sunlight [52]. On top of the decrease in levels of 25(OH)D, elders have a decrease in the renal production of 1,25-dihydroxyvitamin D due to the generalized decline in renal function associated with aging, a decrease in intestinal calcium absorption, resistance to the action of 1,25(OH)D in the bowel, and a decrease in the number of cellular receptors of vitamin D (VDR) [26]. Furthermore, elders are more likely to take medications that may interfere with vitamin D metabolism [53].

We recognize that one limitation of this study is the small number of subjects included. However, a study that analyzed the causal relation between vitamin D status and obesity within 21 cohorts (42,024 subjects) agreed with our data, finding that for every increase of 1 kg/m^2^, there was a reduction of 1.15% of serum 25(OH)D. Here, we found a 1.27% reduction associated to BMI in our population. In this remarkable work, the single nucleotide polymorphism that they chose as marker for obesity was related to an increase in BMI and a reduction in 25(OH)D, reinforcing the idea that obesity causes vitamin D serum levels to drop but that vitamin D status does not affect BMI [54]. Regarding the potential mechanism to explain the association between obesity, BMI, and body fat, vitamin D deficiency is determined less by intestinal absorption and more by sequestration by adipose tissue [55–57]. Some others have indicated that the association between obesity and vitamin D deficiency may be explained almost completely by a simple dilutional effect [46]. It has been also observed that obese people have a 57% lower increase in vitamin D in response to UVB radiation or oral supplementation [23]. Adipose cells have VDR and thus may be influenced by vitamin D status [58].

### 4.3. Inflammatory Status

Finally, given the crescent data that correlates vitamin D deficiency with many diseases, we performed an analysis of the inflammatory status of these subjects by analyzing their serum cytokine profile. We found that IL-1*β*, IL-6, IL-10, IL-18, MCP-1, and TNF-*α* significantly varied with the season, considering age, sex, and individual as random effects, with the highest levels occurring during autumn and/or winter, and the lowest levels occurring in the spring.

However, TNF-*α* was the only cytokine that was dependent on 25(OH)D concentrations. TNF-*α* levels were exacerbated in subjects with vitamin D deficiency. This demonstrates that vitamin D modulation by the inflammatory system depends on the immune system activation for the signaling pathway to allow VDR and vitamin D regulators like CyP27B1 to be expressed. Taking this into consideration, we ensured that all subjects included in this study were healthy. We were able to perform an analysis that correlated perfectly with the cytokine families and their influences between each other, validating our data. Furthermore, in agreement with our data, Peterson and Heffernan reported a negative correlation between vitamin D and TNF-*α* levels in healthy Caucasian women (aged 25–62), a relationship that remained after controlling body fat percentage, menopausal status, age, serum estradiol, serum cortisol, and hormonal contraceptive use [10]. Others found no relationship with IL-6 or IL-10 [6].

Alike our study, another study performed in younger women (aged 19–47) from Kuwait analyzed a wide spectrum of cytokines (IL-1 *β*, IL-6, IL-8, IL-17, IFN-*γ*, TNF-*α*, IL-4, IL-10, and IL-13) and only found a negative correlation between deficient levels of 25(OH)D with TNF-*α* when C-reactive protein (CRP) was elevated [59]. Moreover, a cross-sectional study in adults (aged 20–59) in Brazil showed a negative association between plasma IL-6 and TNF-*α* levels and vitamin D in normal-weight participants [60]. A very recent analysis in three studies within the Human Functional Genomics Project that also supports our data found that despite the impact of seasonality on cytokine production, there is indeed a limited dependency on vitamin D levels [61].

Regarding aging, one study reported an increase of IL-4-producing CD4^+^ cells and a decline in TNF-*α* and interferon-*γ*-producing CD4^+^ cells in older adults [62]. However, another study showed naïve, cytotoxic, and memory CD8^+^ T cells increased the production of type 1 cytokines (interferon-*γ*, TNF-*α*, and IL-2) with age. There was also an increase with aging in the memory CD8^+^ T cell-producing type 2 cytokines (IL-4, IL-6, and IL-10) [63].

For older adults, several studies are in line with the relation between the inflammatory status and 25(OH)D levels found in the present study. For instance, Liu et al. reported that 25(OH)D 1alpha hydroxylase knockout (CYP27b1−/−) mice, when compared with wild-type mice, had more DNA damage, reactive oxygen species production, inflammatory infiltration of the colon, and production of inflammatory cytokines related with SASP [64]. Accordingly, a previous report demonstrates that 1,25(OH)2D deficiency causes erosion of articular cartilage by inducing DNA damage and the production of senescence-associated inflammatory cytokines [65]. Studies in humans have shown that vitamin D deficiency has also been linked with telomere shortening [66, 67]. Telomere shortening seen in women with 25(OH)D < 16 ng/mL (lowest tertile) was equivalent to 5 years of cellular aging when compared with the telomere length of women with >50 ng/mL (highest tertile) [66]. The high prevalence of vitamin D in our population may thus have implications regarding the production of SASP inflammatory cytokines, mainly TNF-*α*.

Finally, recent research suggests that vitamin D exhibits anti-inflammatory effects that might contribute to its beneficial impact on several chronic diseases such autoimmune disorders, osteoarthritis, or cancer [10–12]. For instance, studies in cancer cells reveal that vitamin D regulates several of the key molecular pathways involved in procarcinogenesis inflammation such as prostacyclin synthesis, activation of kinase signaling, and nuclear factors involved in angiogenesis [68]. Our findings suggest that the high prevalence of vitamin D deficiency might place our population at increased risk for a variety of chronic inflammatory diseases. Thus, strategies to increase levels of 25(OH)D levels could translate into a better prognosis and improve the outcomes in the older adult population.

## 5. Conclusions

In this 12-month follow-up study in older individuals, 25(OH)D levels were below 30 ng/mL, and the prevalence of deficiency was high through all four seasons, although it was higher in winter than in autumn and summer, despite the fact that the study took place in a region with a latitude that provides adequate sunlight in all seasons except winter. 25(OH)D levels showed a negative correlation with BMI, WC, and weight. These anthropometric markers are predictors of vitamin D deficiency. A BMI > 32 and a WC > 88 cm can predict a 90% probability of vitamin D deficiency, independent of age, with a confidence level of 73%. When BMI is <32.7, age is a determinant of vitamin D status, with a cutoff point of 74 years. Given the prediction value, these anthropometric and age parameters allow us to generate a simple way to predict vitamin D deficiency that could aid clinicians in better approaching the management of patients with diseases that are associated with this condition. More studies are needed, especially in regions near the equator, to corroborate our results and to elucidate potential mechanisms for such an extraordinary vitamin D deficiency.

## Figures and Tables

**Table 1:** Anthropometric parameters.

Parameter	Mean (range)
Age (years)	68.8 (55–86)
Sex (F/M)	F (13) M (10)
Skin (I–VI)	II (1) III (4) IV (17) V (1)
Smoker (Y/N)	Yes (4) No (19)
Cigarettes per day	1.3 (0–20)
Vitamin D (ng/mL)	20.7 (5–41.5)
Weight (kg)	77.7 (54.8–102.1)
Height (m)	1.64 (1.48–1.81)
Fat (%)	35.2 (17.5–51.3)
Waist (cm)	99.8 (78–121)
BMI (kg/m^2^)	29 (20.1–46.6)
Vitamin D intake (IU)	112 (0–391.4)
Sun exposure (min)	82.3 (0–840)
PTH (pg/mL)	83.7 (21.8–282.3)
Calcium (pg/mL)	9.0 (7.6–10.1)

**Table 2:** Stepwise regression of correlated parameters versus 25(OH)D level serum concentration, in ng/mL.

Variable	Effect and significance (B/p)		
	Model		
	1	2	3
Adjusted *R*^2^	0.27	0.32	0.31
Waist (cm)	−0.38/2.8 × 10–7	−0.18/0.09	−0.20/0.07
BMI (kg/m^2^)	—	−0.53/0.01	−0.65/0.02
Weight (kg)	—	—	0.08/0.50

**Table 3:** Confusion table. Predicted versus actual 25(OH)D levels.

Original	Predicted			
	Deficient	Insufficient	Sufficient	Recall
Deficient	40	5	0	88.9%
Insufficient	9	23	7	59.0%
Sufficient	0	4	7	63.6%
Precision	81.6%	71.9%	50.0%	73.7%

Predicted vitamin D levels results after following the decision tree in Figure 3 and actual vitamin D status (rows) of the patients (women) in this study.
